# Postpartum blood pressure trends and time in target range during remote blood pressure monitoring

**DOI:** 10.1002/pmf2.70470

**Published:** 2026-10-06

**Authors:** Sarah Shim, Katelyn M. Tessier, Sarah A. Wernimont, Tiffany Corlin, Jessica L. Gottula, Martina Burn, Bethany A. Sabol

**Affiliations:** ^1^ Department of Obstetrics Gynecology and Women's Health University of Minnesota Minneapolis Minnesota USA; ^2^ Biostatistics Core Masonic Cancer Center University of Minnesota Minneapolis Minnesota USA; ^3^ Division of Maternal‐Fetal Medicine Department of Obstetrics Gynecology and Women's Health University of Minnesota Minneapolis Minnesota USA

**Keywords:** hypertension, hypertensive disorder of pregnancy, maternal morbidity, postpartum, preeclampsia, remote blood pressure monitoring

## Abstract

**Introduction:**

Postpartum remote blood pressure monitoring (RBPM) programs have emerged as an important adjunct to the management of hypertensive disorders of pregnancy (HDP). These programs have demonstrated robust patient participation and satisfaction with reduced readmissions and severe maternal morbidity. Despite this, data describing blood pressure (BP) trends throughout program participation and across different antihypertensive regimens remain limited. We sought to evaluate BP trends and time in target range (TTR) during our 6‐week RBPM program.

**Methods:**

This is a retrospective cohort study of postpartum individuals participating in an RBPM program from November 2023 through December 2024. This program utilizes a standardized nurse (RN)‐driven protocol for antihypertensive medication initiation and titration to achieve a goal BP of <140/90 mmHg. Target range was defined as SBP (systolic BP) 100–139 mmHg and DBP (diastolic BP) 60–89 mmHg. The primary outcomes were BP trend and TTR throughout program participation stratified by antihypertensive medication at discharge.

**Results:**

The cohort included 465 individuals. Mean BPs were <140/90 mmHg for all groups for each program week. Most participants were within the target range throughout with TTR between 80.3% and 90.4%. Of participants, 175 (81.4%) were prescribed nifedipine monotherapy and 40 (18.6%) labetalol monotherapy. Mean BP trends were within goal for both groups; however, those discharged with nifedipine spent more TTR (nifedipine 87.5% vs. labetalol 80.3%, *p* = 0.03). There were no significant differences in rates of severe hypertension or hypotension; however, those on nifedipine experienced fewer elevated BPs and fewer patients who were discharged on nifedipine required antihypertensives at 6 weeks postpartum (nifedipine 35.7% vs. labetalol 73.5%, *p* < 0.001; adjusted odds ratio [OR], 0.27; 95% confidence interval [CI], 0.11–0.61).

**Conclusion:**

Postpartum RBPM is an important tool for postpartum care. Utilization of a standardized RN‐driven protocol for antihypertensive medication initiation and titration can result in optimal BP management irrespective of medication regimen at discharge. These data suggest that nifedipine may be associated with more rapid and sustained achievement of target BPs throughout 6 weeks postpartum compared to labetalol.

## 1 INTRODUCTION

Hypertensive disorders of pregnancy (HDP) are a group of hypertension‐related disorders occurring both during pregnancy and following birth, affecting 2%–8% of birthing people [1]. They include both pre‐gestational or chronic hypertension and pregnancy‐associated hypertensive disorders such as gestational hypertension, preeclampsia with and without severe features, eclampsia, and Hemolysis, Elevated Liver Enzymes, and Low Platelets (HELLP) syndrome [2]. These diagnoses exist on a spectrum of disease and continue to be a leading cause of maternal morbidity and mortality. Postpartum physiology results in peak blood pressures occurring 3–6 days following birth, the timing of which often occurs after hospital discharge, when vigilance is lower and prompt treatment is less accessible [3, 4]. These factors contribute to the postpartum period being one of the highest risk times for severe hypertension, maternal complications such as stroke, and death, particularly if there is a delay in recognition and treatment [5, 6, 7].

Postpartum remote blood pressure monitoring (RBPM) programs have emerged as a novel way to enhance surveillance and have been demonstrated to be a safe, feasible, and efficacious way to improve blood pressure management and reduce hospital readmission and severe maternal morbidity [8, 9, 10, 11, 12, 13, 14, 15]. Despite these successes, there remains wide variation in program structure with a lack of robust data on blood pressure trends during program participation, optimal strategies to maintain blood pressure in goal range, and the most effective medications to utilize [16, 17, 18, 19]. This study aims to evaluate how a postpartum RBPM program in conjunction with a standardized approach to blood pressure management influences postpartum blood pressure trends and blood pressure metrics. We additionally explore the impact of specific antihypertensive agents at discharge on these clinical outcomes.

## 2 MATERIALS AND METHODS

### 2.1 Postpartum RBPM program description

Home Observation of Postpartum Elevated Blood Pressure (HOPE‐BP) is our institution's postpartum RBPM program for birthing people with HDP. Upon hospital discharge, individuals with HDP who are English‐speaking and have access to the patient portal of our hospital's electronic health record (EHR) are recommended to enroll. They are provided a correctly sized blood pressure cuff and standard education on how to take a home blood pressure. Once home, they are instructed to provide twice daily remote blood pressure values that are uploaded to MyChart care companion in our systems’ EHR, Epic. When a blood pressure reading is submitted, patients receive automated and immediate feedback on their blood pressure value. Blood pressures are monitored Monday–Friday during business hours (8 am–4 pm) by our dedicated HOPE‐BP nursing team. Under physician supervision, coupled with a nursing standing order set and standardized medication algorithm, antihypertensive medications can be initiated and titrated to maintain optimal blood pressure. Blood pressure goals for this program were developed to align with the American Heart Association and American College of Cardiology 2017 Guidelines, targeting lower thresholds in the postpartum period with a goal of keeping blood pressures less than 140 mmHg systolic and 90 mmHg diastolic [20]. Blood pressures above this goal on two or more occasions prompt initiation or titration of antihypertensives (Supporting Information).

### 2.2 Study design

We performed a retrospective cohort study of postpartum individuals with an HDP discharged following delivery at the University of Minnesota Medical Center and who participated in our institution's RBPM program HOPE‐BP from November 2023 through December 2024.

Only patients eligible to participate in HOPE‐BP were included. Inclusion criteria for HOPE‐BP required an HDP diagnosis prior to or during their birth admission (as defined by the American College of Obstetricians and Gynecologists guidelines), English as a preferred language, and access to MyChart Care Companion [21, 22]. Eligible patients were identified using the HOPE‐BP order within the EHR. Of eligible patients, those who opted out of research were excluded.

The study adhered to Strengthening the Reporting of Observational Studies in Epidemiology (STROBE) guidelines and was approved by the Institutional Review Board of the University of Minnesota (IRB#: STUDY00024251). Maternal demographic and clinical information were obtained directly from manual chart abstraction and by utilizing the University of Minnesota Obstetric Measures (UMOM) Database. The UMOM Database (Wernimont SA, Principal Investigator IRB# STUDY00012822) is a clinical outcomes database that links to data found within the EHR from nine birthing hospitals within the MHealth Fairview system from 2011 until present.

The primary outcomes of this study were (1) overall blood pressure trend and (2) time in target range (TTR). These outcomes were stratified by antihypertensive medication at discharge. Overall blood pressure trend was defined as the average systolic and diastolic blood pressures (SBP and DBP) by program week for all blood pressures entered into the home monitoring program through 6 weeks postpartum. TTR for each participant was defined as an SBP of 100–139 mmHg and a DBP of 60–89 mmHg. TTR was calculated using the Rosendaal method [23]. By assuming a linear change between consecutive measurements, this method interpolates values for the time between each known measurement, with elapsed time measured continuously to the minute rather than in discrete hourly or daily units. Then, the proportion of total time that values fall within the target range was calculated. Outside of the target range, blood pressures were additionally categorized as being low (defined as SBP <100 mmHg or DBP <60 mmHg), elevated (defined as SBP range of 140–159 mmHg or DBP range of 90–109 mmHg), and severe (defined as SBP ≥160 mmHg or DBP ≥110 mmHg). To be considered in the target range, both SBP and DBP had to fall within these parameters. For the other value ranges, if either SBP or DBP fell within the range, it was categorized as such with classification always erring on the side of the out‐of‐range categorization. For example, a blood pressure of 99/65 mmHg would be categorized as low whereas a blood pressure of 135/90 mmHg would be categorized as elevated. Participants who did not provide ≥2 blood pressures were excluded from TTR analysis.

Overall blood pressure trend and TTR were stratified by antihypertensive medication at discharge for all participating patients. A subgroup analysis was performed to specifically compare our primary outcomes between those discharging on labetalol versus nifedipine. Secondary outcomes for this analysis included postpartum readmission, antihypertensive medication changes or dose escalation, 6‐week postpartum clinic appointment attendance, SBP and DBP values at the 6‐week postpartum visit, and need for continued antihypertensive management at 6 weeks postpartum.

### 2.3 Statistical analysis

Descriptive statistics summarized demographic and clinical characteristics using frequency and percentage for categorical variables and mean and standard deviation (SD) for continuous variables. Categorical variables were compared by antihypertensive medication at discharge using Chi‐square or Fisher's exact tests; Student's *t*‐tests were used for continuous variables. Similarly, primary and secondary outcomes were analyzed using Student's *t*‐tests, Chi‐square, or Fisher's exact tests. Adjusted analyses (for last inpatient SBP, gestational age at birth, parity and chronic hypertension) were performed using linear and logistic regression. Mean difference or odds ratio (OR) and 95% confidence intervals (CIs) were obtained. All reported *p* values were two‐sided and a significance level of 0.05 was used. Statistical analyses were performed using R (version 4.6.0, R Core Team).

## 3 RESULTS

From November 2023 to December 2024, there were a total of 699 individuals enrolled in the HOPE‐BP program. Of those, 464 (66.4%) were included for analysis (Figure 1). Participants who did not enroll or participate in the program (*n* = 99) differed significantly from those included in the analysis (*n* = 464) with respect to race (*p* < 0.001), insurance status (*p* < 0.001), and parity (*p* = 0.021); shown in Table S1. Excluded participants were more likely to identify as Black or American Indian/Alaska Native, to have public insurance, and to be multiparous, compared to included participants. Maternal age, gestational age at birth, and pre‐gravid body mass index did not differ significantly between groups. Baseline characteristics for included participants are represented in Table 1. The mean maternal age at birth was 32 years (±5.6) with most births occurring at term (mean gestational age at birth of 37.3 ± 3.2 weeks) to nulliparous (55.2%), white (68.5%), and patients with private insurance (60.8%). The most common HDP diagnosis was gestational hypertension (*n* = 237, 51.2%) followed by preeclampsia with severe features (*n* = 137, 29.6%). The majority of patients were discharged on nifedipine (*N* = 175, 37.7%) or were not discharged on antihypertensive therapy (*N* = 146, 31.5%).

**FIGURE 1:**
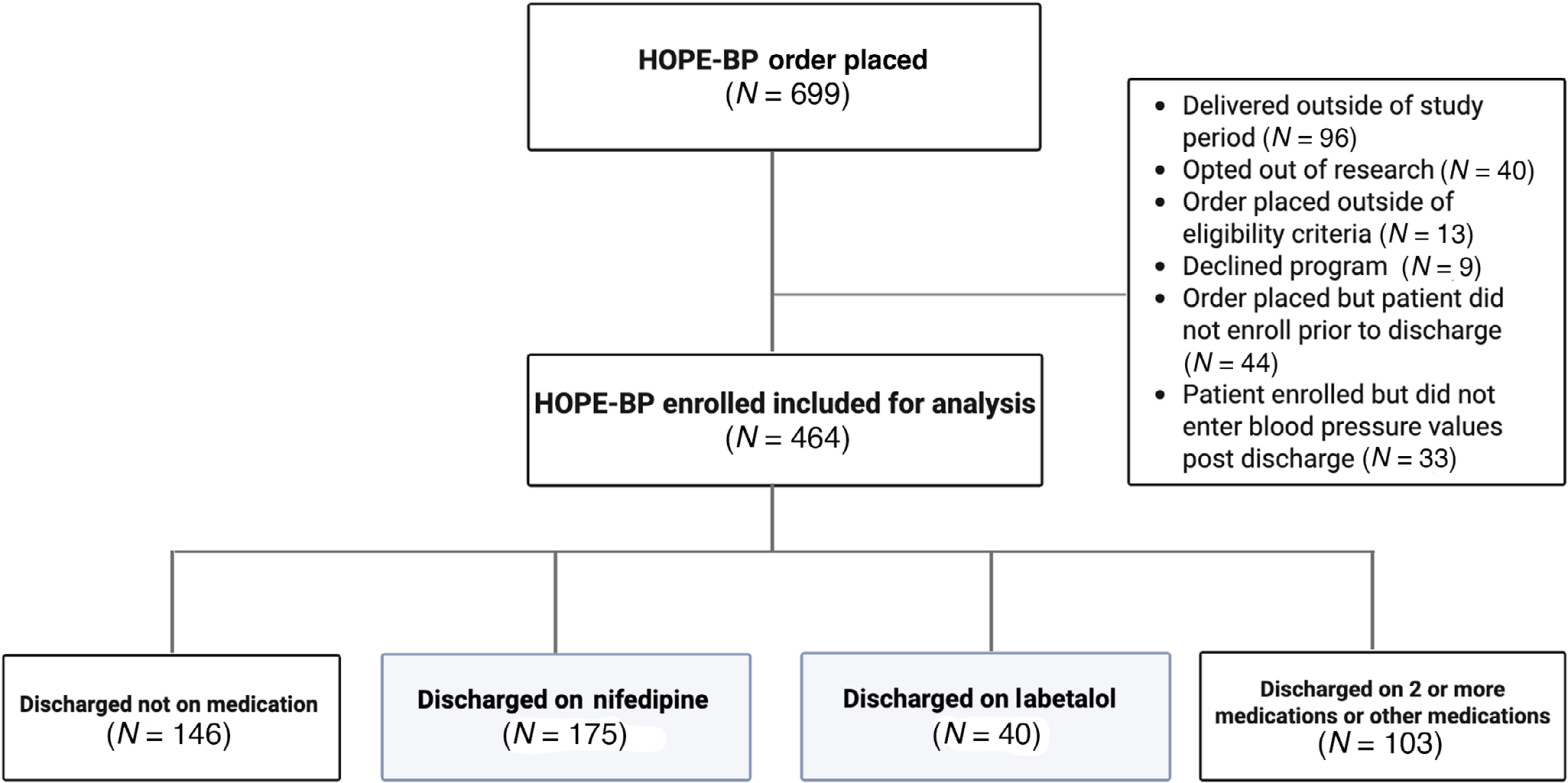
Study flow diagram. HOPE‐BP, Home Observation of Postpartum Elevated Blood Pressure.

**TABLE 1:** Baseline characteristics of HOPE‐BP participants.

	Total (*n* = 464)	No medication (*n* = 146)	2+ medication or other (*n* = 103)	Labetalol (*n* = 40)	Nifedipine (*n* = 175)	*p* value^a^ (labetalol vs. nifedipine)
Maternal age at birth (years)	32.2 (5.6)	31.6 (5.1)	32.5 (6.0)	33.8 (7.0)	32.6 (5.3)	0.073
Gestational age at birth (weeks)	37.3 (3.2)	38.1 (2.7)	35.5 (3.6)	36.7 (3.7)	37.9 (2.6)	0.013
Pre‐gravid BMI^b^ (kg/m^2^)	30.2 (8.1)	29.5 (6.7)	31.8 (8.2)	31.3 (8.1)	29.7 (9.0)	0.431
BMI at birth^c^ (kg/m^2^)	35.0 (8.1)	34.02 (6.3)	36.1 (9.0)	37.5 (9.8)	34.6 (8.3)	0.054
Last inpatient SBP (mmHg)	127.8 (10.3)	123.4 (9.7)	131.5 (10.1)	132.7 (11.1)	128.1 (9.4)	*0.008*
Last inpatient DBP (mmHg)	81.9 (8.1)	79.2 (84.3)	83.6 (8.6)	84.3 (8.7)	82.7 (7.2)	*0.215*
Maximum inpatient SBP (mmHg)	141.9 (12.7)	134.0 (10.9)	150.32 (12.6)	144.2 (10.7)	142.9 (10.8)	*0.514*
Maximum inpatient DBP (mmHg)	91.5 (8.0)	86.8 (6.6)	96.1 (8.0)	91.7 (8.3)	92.7 (7.1)	*0.439*
**Maternal race** ^d^						*0.638*
White	311 (68.7)	98 (71.0)	72 (69.9)	23 (57.5)	118 (68.6)	
Black	78 (17.2)	22 (15.9)	17 (16.5)	10 (25.0)	29 (16.9)	
Asian	37 (8.2)	11 (8.0)	8 (7.8)	5 (12.5)	13 (7.6)	
American Indian or Alaskan Native	6 (1.3)	8 (1.4)	2 (1.9)	0 (0.0)	2 (1.2)	
Native Hawaiian or other Pacific Islander	1 (0.2)	0 (0.0)	0 (0.0)	0 (0.0)	1 (0.6)	
Other (including Multiracial)	20 (4.4)	5 (3.6)	4 (3.9)	2 (5.0)	9 (5.2)	
Nulliparity	256 (55.2)	81 (55.5)	61 (59.2)	15 (37.5)	99 (56.6)	*0.029*
**Insurance status**						*0.527*
Public insurance	174 (37.5)	53 (36.3)	42 (40.8)	17 (42.5)	62 (35.4)	
Private insurance	282 (60.8)	90 (61.6)	59 (57.3)	23 (57.5)	110 (62.9)	
Uninsured	8 (1.7)	3 (2.1)	2 (1.9)	0 (0)	3 (1.7)	
Tobacco use	38 (8.2)	4 (2.7)	13 (12.6)	4 (10.0)	17 (9.7)	*0.956*
**Diabetes**						
No diabetes	384 (82.8)	124 (84.9)	83 (80.6)	31 (77.5)	146 (83.4)	*0.162*
Pregestational diabetes	32 (6.9)	7 (4.8)	12 (11.7)	5 (12.5)	8 (4.6)	
Gestational diabetes	48 (10.3)	15 (10.3)	8 (7.8)	4 (10.0)	21 (12.0)	
**HDP type**						*0.018*
Gestational hypertension	237 (51.2)	115 (78.8)	21 (20.4)	10 (25.0)	91 (52.0)	
Chronic hypertension	60 (13.0)	13 (8.9)	14 (13.6)	12 (30.0)	21 (12.0)	
Preeclampsia without severe features^e^	26 (5.6)	6 (4.1)	6 (5.8)	3 (7.5)	11 (6.3)	
Preeclampsia with severe features^e^	137 (29.6)	11 (7.5)	62 (60.2)	15 (37.5)	49 (28.0)	
HELLP	3 (0.6)	0 (0.0)	0 (0.0)	0 (0.0)	2 (1.1)	
Eclampsia	1 (0.2)	0 (0.0)	0 (0.0)	0 (0.0)	1 (0.6)	
**Delivery type**						*0.287*
Vaginal	195 (41.9)	74 (50.7)	25 (24.3)	17 (42.5)	79 (44.9)	
Cesarean	257 (5545)	70 (47.9)	77 (74.8)	23 (57.5)	87 (49.7)	
Operative birth	12 (2.6)	2 (1.1)	1 (1.0)	0 (0.0)	9 (5.1)	

*Note*: Data are *n* (%), mean (SD).

Abbreviations: BMI, body mass index; DBP, Diastolic Blood Pressure; HDP, hypertensive disorders of pregnancy; HELLP, Hemolysis, Elevated Liver Enzymes, and Low Platelets; SBP, Systolic Blood Pressure.

^a^ To investigate the association between discharge medication and continuous variables, Student's *t*‐tests were used. Chi‐square tests were used for categorical variables.

^b^ Missing for 87 patients; 28 with no medications, 19 with 2+ meds/other, 10 in labetalol only, and 30 in nifedipine only.

^c^ Missing for 8 patients; 4 with no medications, 2 with 2+ meds/other, and 2 patients in nifedipine only.

^d^ Missing for 11 patients; 8 with no medications, and 3 patients in nifedipine only.

^e^ Including superimposed preeclampsia.

The evaluation of overall blood pressure trend for the entire cohort over the course of the 6‐week program demonstrated mean SBP and DBP below 140 and 90 mmHg, respectively, for each program week (Figure 2). The steepest blood pressure decline was observed within the first week of the program, followed by a plateau in both SBP and DBP by Week 4 of the program. When stratified by medication at discharge, the trends observed for the entire cohort persisted. Regardless of medication at discharge, average SBP and DBP were maintained less than 140 and 90 mmHg, respectively, for all groups for each program week.

**FIGURE 2:**
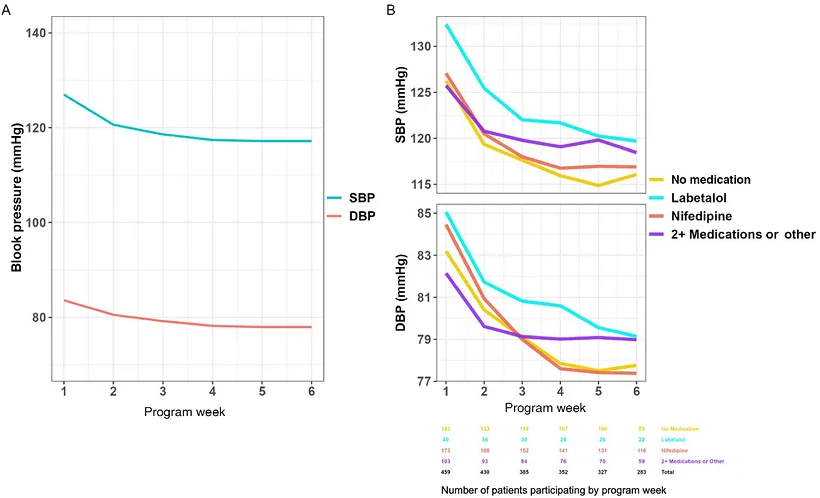
Systolic and diastolic blood pressure (SBP and DBP) trend by program week for all participants (A) including stratification by antihypertensive medication at discharge (B). The number of participants entering at least one blood pressure in each program week by antihypertensive at discharge is represented in the table below panel (B).

Among the 464 eligible participants, the median number of BP readings over the 6‐week follow‐up period was 35 readings (of an expected maximum of 84); 10 participants (2.2%) with fewer than two valid readings could not be interpolated and were also excluded from the TTR analysis. The median interval between consecutive readings used for interpolation was 19.1 h; 79.6% of intervals reflected a gap of 1 day or less, 14.1% were between 1 and 2 days, 3.2% between 2 and 3 days, 1.4% between 3 and 4 days, and 1.7% exceeded 4 days (Table S2). Specifically, when TTR was calculated, regardless of the antihypertensive medication at discharge, the majority of participants were within the target range throughout (TTR by group varied between 80.3% and 90.3%; Table 2). Of the 146 patients who did not require antihypertensive medication at discharge, 42 (28.8%) were ultimately started on antihypertensives during the program, with 90.5% (*n* = 38) being initiated on nifedipine. Episodes of severe hypertension and hypotension were rare for all patients (Table 2).

**TABLE 2:** Blood pressure control represented by time in target range (TTR) stratified by antihypertensive medication at discharge

	Total (*n* = 464)	No medication^a^ (*n* = 146)	2+ Medications or other^b^ (*n* = 103)	Labetalol (*n* = 40)	Nifedipine (*n* = 175)	*p*‐value^c^ (labetalol vs nifedipine)	Mean difference (95% CI)	*p*‐value^d^
In range	87.5 (16.8)	90.3 (15.0)	86.2 (16.5)	80.3 (22.4)	87.5 (16.6)	*0.026*	5.07 (−1.56, 11.7)	*0.133*
Elevated	10.3 (16.0)	7.0 (13.1)	11.6 (15.8)	18.3 (22.4)	10.6 (15.9)	*0.014*	−5.28 (−11.7, 1.13)	*0.106*
Severe	0.2 (1.3)	0.2 (1.5)	0.2 (0.8)	0.2 (1.0)	0.2 (1.3)	*0.829*	0.02 (−0.45, 0.49)	*0.919*
Low	2.0 (6.0)	2.4 (1.2)	2.0 (4.7)	1.2 (3.2)	1.8 (5.7)	*0.536*	0.18 (−1.81, 2.17)	*0.857*

*Note*: Data are mean (SD).

Abbreviation: CI, confidence interval.

^a^ Of these patients, 42 were initiated on antihypertensive at some point during the six‐week program; 90.5% (*n* = 38) of whom were initiated on nifedipine.

^b^ 1 agent: enalapril monotherapy (*n* = 5) or other monotherapy (*n* = 5); 2 agents: labetalol + nifedipine (*n* = 48), nifedipine + hydrochlorothiazide (HCTZ) (*n* = 14), labetalol + HCTZ (*n* = 11); 3 agents: combinations of labetalol, nifedipine, HCTZ, enalapril, and/or other (*n* = 20); 4 agents: labetalol, nifedipine, HCTZ, and other (*n* = 1)

^c^ To investigate the association between time in range and antihypertensive medication at discharge, Student's *t*‐tests were used.

^d^ Adjusted linear regression models were used to adjust for last inpatient SBP, GA at birth, parity, and chronic hypertension. Mean difference and 95% confidence intervals are presented.

A subgroup analysis of patients discharging on labetalol compared to nifedipine was then performed from the same study cohort (*N* = 215). Of the 215 included, the majority were prescribed nifedipine monotherapy (*n* = 175, 81.4%) rather than labetalol monotherapy (*n* = 40, 18.6%). Baseline characteristics between the labetalol versus nifedipine monotherapy groups were similar (Table 1). Both groups had a mean gestational age approximately at term, although those discharging on labetalol gave birth slightly earlier (36.7 weeks vs. 37.9 weeks, *p* = 0.013). Those discharging on nifedipine were more likely to be nulliparous (56.6% vs. 37.5%, *p* = 0.029). The most common hypertensive diagnosis for those discharging on labetalol was preeclampsia with severe features (*n* = 15, 37.5%), whereas the most common diagnosis for those discharging on nifedipine was gestational hypertension (*n* = 91, 52.0%). Regardless of medication at discharge (labetalol or nifedipine), the average systolic and diastolic blood pressures for each week in the program were within goal; however, participants on nifedipine exhibited lower mean SBP and DBP during each program week (Figure 3). Those discharged on nifedipine spent significantly more TTR compared to those discharged on labetalol (87.5% vs. 80.3%,*p* = 0.026). In addition, those discharged on nifedipine experienced less time in the elevated range (10.6% vs. 18.3%, *p* = 0.014). There were no significant differences in the rates of severe hyper‐ or hypotension between the two groups (Table 2, Figure 3).

**FIGURE 3:**
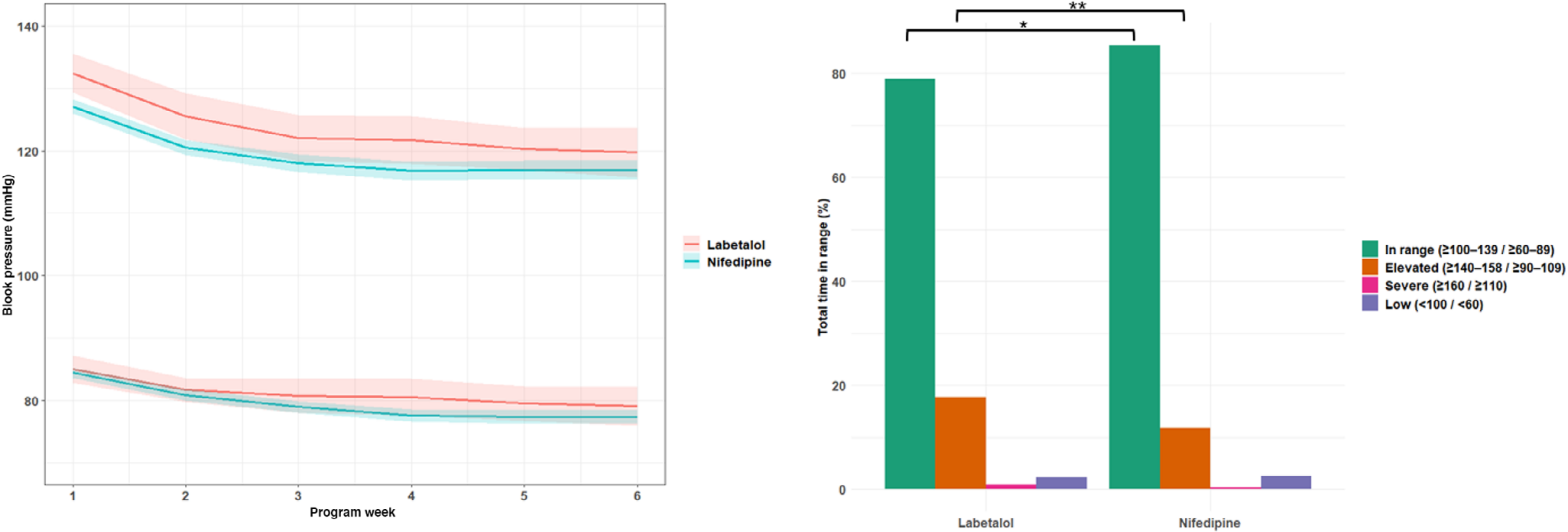
Blood pressure (BP) trend (left) and total time in target range (TTR) (right) during postpartum RBPM in patients discharging on nifedipine compared to labetalol. RBPM, remote blood pressure monitoring. ^*^ *p* = 0.026; ^**^ *p* = 0.014.

A larger percentage of patients discharging on labetalol required one or more dose escalations compared to those discharging on nifedipine (any escalation: 45% vs. 28.6%, *p* = 0.044; more than one escalation: 30.0% vs. 14.9%, *p* = 0.071). Patients discharging on labetalol also had higher average SBPs at 6 weeks postpartum (mean [SD]: 124.5 [12.2] vs. 119.2 [11.0], *p* = 0.025). However, these associations were non‐significant in the adjusted analysis. There were no differences in postpartum visit attendance rates or DBPs at 6 weeks postpartum in both unadjusted and adjusted analyses (Table 3). Fewer individuals who discharged on nifedipine remained on antihypertensive medications at 6 weeks postpartum compared to individuals discharged on labetalol (35.7% vs. 73.5%, *p* < 0.001; adjusted OR, 0.27; 95% CI, 0.11–0.61). While the percentage of readmissions was less in the nifedipine group compared to labetalol, this difference did not reach statistical significance (10.0% vs. 5.7%, *p* = 0.301).

**TABLE 3:** Association between secondary outcomes and discharge medication.

	Labetalol (*n* = 40)	Nifedipine (*n* = 175)	*p*‐value^a^	Estimate (95% CI)^b^	*p*‐value^b^
Postpartum visit attendance	29 (72.5)	137 (78.3)	*0.431*	1.16 (0.48, 2.68)	*0.737*
Systolic blood pressure at 6 weeks postpartum (mmHg)	124.5 (12.2)	119.2 (11.0)	*0.025*	−3.03 (−7.83, 1.76)	*0.213*
Diastolic blood pressure at 6 weeks postpartum (mmHg)	80.1 (7.5)	77.9 (7.9)	*0.172*	−1.65 (−5.06, 1.76)	*0.340*
Antihypertensive at 6 weeks postpartum^c^	25 (73.5)	56 (35.7)	*<0.001*	0.27 (0.11, 0.61)	*0.003*
Postpartum readmission	4 (10.0)	10 (5.7)	*0.301*	—	—
Need for antihypertensive dose escalation	18 (45.0)	50 (28.6)	*0.044*	0.62 (0.29, 1.34)	*0.217*
Number of antihypertensive dose escalations needed			*0.071*	—	—
0	22 (50.0)	125 (71.4)			
1	6 (15.0)	24 (13.7)			
>1	12 (30.0)	26 (14.9)			

*Note*: Data are *n* (%), mean (SD).

Abbreviation CI, confidence interval.

^a^ Student's *t*‐tests were used for continuous variables; Chi‐square or Fisher's exact tests were used for categorical variables.

^b^ Adjusted regression models were used to adjust for last inpatient SBP, GA at birth, parity, and chronic hypertension. Linear regression was used for continuous outcomes, and mean difference and 95% confidence intervals are presented. Logistic regression was used for binary outcomes, and odds ratio and 95% confidence intervals are presented.

^c^ Missing for 24 patients; 6 on labetalol only and 18 on nifedipine only.

## 4 DISCUSSION

This study adds to the literature supporting the use of RBPM programs in postpartum surveillance, demonstrating that an approach which combines lower blood pressure parameters for initiation and titration of antihypertensives in combination with nurse‐driven surveillance and monitoring protocols can lead to optimal blood pressure management regardless of antihypertensive at discharge, defined as SBP 100–139 mmHg and DBP 60–89 mmHg [20]. Using this protocolized approach, this cohort illustrates that the majority of participants achieved blood pressures within the goal range throughout the entire 6‐week postpartum period. Additionally, existing studies have displayed the benefit of RBPM by identifying lower blood pressures at isolated occurrences (i.e., 6‐week postpartum visit, 12 months postpartum, etc.) [24, 25]. However, by representing blood pressure through a calculation of TTR (a well‐established method and considered standard way of reporting BP control in the non‐pregnant hypertension literature), we have introduced an innovative approach to the analysis of postpartum RBPM values to better represent the duration of optimal blood pressures as opposed to traditional representations of blood pressure trajectories throughout our entire 6‐week postpartum program [26].

The addition of our subgroup analysis comparing TTR between labetalol and nifedipine adds important information to the postpartum literature on optimal medication. Labetalol and nifedipine are both first‐line antihypertensives recommended by the American College of Obstetricians and Gynecologists for use in pregnancy and the postpartum period for management of HDP [21]. Recent studies have sought to compare outcomes for postpartum individuals with HDP discharged on nifedipine versus labetalol with the aim of identifying if one antihypertensive agent is superior. Several studies have identified a higher rate of postpartum readmission in patients discharged on labetalol compared with nifedipine [16, 19, 27]. However, these studies analyze blood pressure values obtained only in the hospital setting with limited data comparing overall postpartum blood pressure trends in the 6‐week postpartum period. Additionally, outside of hospital readmission, other outcomes such as requirement of antihypertensive medication initiations or escalations following discharge have not been studied.

While existing literature is suggestive that nifedipine may be superior to labetalol in reducing rates of readmission after discharge, the data presented here provide more comprehensive information on blood pressure trends throughout the entirety of the 6‐week postpartum period based on antihypertensive medication at discharge [16, 18, 19, 27]. A recent study found that discharge on labetalol compared with nifedipine was associated with more severe hypertension on home BP monitoring in the first 10 days postpartum which they postulated may drive increased readmission rates in those discharged on labetalol [28]. Consistent with these findings, our study found that when compared to individuals discharged on labetalol, individuals discharged on nifedipine spent more time in the initial postpartum period with blood pressures in target range. While our study was not designed to determine the mechanism underlying the difference in TTR between labetalol and nifedipine groups, several factors may contribute to the improved blood pressure control we observed with nifedipine. First, it is important to acknowledge that the baseline differences in disease severity between groups may partially explain this finding. Patients discharged on labetalol in our cohort were more likely to have preeclampsia with severe features and delivered at an earlier gestational age compared to those discharged on nifedipine, suggesting labetalol may have been preferentially selected for a higher‐acuity population with more severe or refractory hypertension. This differential baseline risk, rather than the medication itself, may account for some of the observed disparity in postpartum blood pressure control.

Aside from possible study design contributions, we hypothesize that dosing regimen and adherence may play a role in the more favorable TTR for nifedipine over labetalol. Nifedipine is dosed once or twice daily, whereas labetalol is often prescribed two to three times daily; it has been suggested that the consistency and ease of a once‐daily regimen may underlie the improved postpartum blood pressure outcomes seen with nifedipine. A simpler regimen may be particularly relevant in the early postpartum period, when patients have competing demands that affect medication adherence [29]. Additionally, a pharmacologic mechanism specific to nifedipine may contribute to more effective blood pressure control. Nifedipine has been associated with improved postpartum diuresis compared with labetalol in prior work, offering a potential biologic mechanism by which it may improve postpartum blood pressure outcomes given that blood pressure control is closely tied to fluid shifts and volume status in the postpartum period [30].

Our study, acknowledging the limitations of its retrospective nature, seemingly adds to the previous work in supporting nifedipine over labetalol as the preferred antihypertensive agent to achieve rapid and sustained target blood pressures throughout the initial 6‐week postpartum period. However, any conclusions regarding the relative contributions of cohort severity, dosing convenience, and underlying pharmacology to the TTR difference observed in this study cannot be definitively known given the retrospective design, and this remains an important area for future prospective investigation.

This study has several strengths. It combines a significant amount of blood pressure data throughout the 6‐week postpartum period with manual chart review performed by a physician to improve the reliability of the outcomes being studied. Additionally, this is a pragmatic evaluation of a current program. To our knowledge, this is one of the first applications of TTR as a metric in the postpartum population with HDP. TTR is increasingly favored in the broader hypertension literature as a standardized way to characterize blood pressure control over time, offering a more clinically meaningful representation of blood pressure recovery than isolated point measurements. This approach lends itself particularly well to the postpartum period, where the 6‐week visit serves as a well‐established clinical endpoint against which blood pressure control is meaningfully assessed. Additionally, this study represents a pragmatic, real‐world RBPM program rather than a controlled experimental intervention. As such, our findings reflect blood pressure control as it is actually achieved in routine clinical practice, including the variability in patient engagement/enrollment, medication titration, and follow‐up that characterizes real‐world program delivery and may therefore be more directly generalizable and guide expectations for RBPM programs.

Our study, however, is not without limitations. Participants who did not complete the program differed demographically from those who did (Table S1), with disproportionately lower completion among Black and American Indian/Alaska Native individuals and those with public insurance. This raises the possibility that our findings may not generalize equally across all populations served by this program, and suggests that barriers to sustained engagement with remote postpartum BP monitoring may not be equally distributed. Future work should prioritize understanding and addressing these barriers to ensure that RBPM programs reach and retain the patients who may benefit most. Furthermore, this was a single‐center study, which may also limit generalizability. Lastly, this study focused on labetalol and nifedipine as the primary antihypertensive agents used within our standardized postpartum treatment algorithm. As evidence and clinical practice for postpartum hypertension management continue to expand beyond first‐line agents to include additional antihypertensive agents (such as diuretics and angiotensin‐converting enzyme inhibitors, for example), future studies incorporating a broader range of antihypertensive regimens will be important to further characterize their impact on postpartum blood pressure recovery.

The study was retrospective in nature and included a relatively small number of study participants. Despite this limitation, robust analyses were performed and as the program amasses more participants, this study can be repeated and inclusion criteria can be expanded to further validate these findings.

## 5 CONCLUSION

RBPM is an important tool for postpartum care. Combining these programs with nurse‐driven standardized protocols for antihypertensive medication initiation and titration can result in optimal blood pressure management irrespective of medication regimen at discharge. Moreover, these data suggest that nifedipine may be associated with more rapid and sustained achievement of target blood pressures throughout 6 weeks postpartum compared to labetalol. Although we recognize that this was not a randomized controlled trial and level 1 evidence is needed to more definitively establish the preferred first‐line antihypertensive agent in the postpartum setting.

## CONFLICT OF INTEREST STATEMENT

The authors declare no conflicts of interest.

## ETHICS STATEMENT

This study was approved by the Institutional Review Board of the University of Minnesota (IRB#: STUDY00024251) utilizing the University of Minnesota Obstetric Measures (UMOM) Database (Wernimont SA, Principal Investigator IRB# STUDY00012822). Our large clinical database contains only individuals who do not opt out of research, and all data were deidentified in analysis.

## Supporting information



Supporting Information

## Data Availability

Data are available upon reasonable request. The datasets generated during and/or analyzed in the current study are available from the corresponding author on reasonable request.
