# Supplementary material for: Postpartum blood pressure trends and time in target range during remote blood pressure monitoring
Source: Pregnancy (Hoboken). 2026 Oct 6;2(6):e70470. doi: 10.1002/pmf2.70470 (PMC13640052; doi:10.1002/pmf2.70470)
Supplement: Supplementary file 1 [file PMF2-2-e70470-s001.docx]

**Supplemental Materials:**

Remote Blood Pressure Monitoring Standardized Medication Management Algorithm

**Supplemental Materials A:**

RBPM Standardized Medication Algorithm for Initiation and Up-Titration of Antihypertensive Medications

Applies to: patients on antihypertensive medications with uncontrolled HTN or not on antihypertensive medications with uncontrolled HTN. Defined as BP ≥140/90 at least 2 consecutive BPs ≥ 6 hours apart OR BP ≥150/100 with other BPs ≥ 130/80.

Development of persistent headache, severe abdominal pain, shortness of breath, chest pain, or vision changes:

Consider need for ER or Provider Office Visit

SBP ≥ 140

-OR-

DBP ≥ 90

*Contact provider to initiate

What is the HR?

< 70 bpm – Start Nifedipine

≥ 70 bpm – Start Labetalol

Consider Enalapril if chronic hypertension and/or pregestational diabetes

Repeat BP X 1

SBP<140 and DBP<90

SBP≥140 or DBP≥90

Start* or Increase Medication

Continue current medication doses

Start Enalapril

5mg daily

Start Labetalol

200mg BID

Start Nifedipine

30 mg daily

30mg

200 mg TID*

If taking another dose BID increase to TID

10 mg BID

60 mg daily

15 mg BID

90 mg daily

Increase by 200 mg increments until 800mg TID

20 mg BID

60 mg BID

Maintain SBP ≤ 140 & DBP ≤ 90; contact provider if max dose reached and in need of 2^nd^ medication

Do not titrate medication more than once in 24 hours

**Supplemental Materials B:**

RBPM Standardized Medication Algorithm Down-Titration of Antihypertensive Medications

START DECREASING ANTIHYPERTENSIVES IF:

BP < 100/60 -or- if patient exhibits symptoms such as lightheadedness, dizziness, weakness, shortness of breath/heavy chest AND is <120/80.

*If weeks 3-6 and < 120/80 mmHg okay to start decreasing per protocol.

DO NOT down titrate with the intention to discontinue medication if the patient has an underlying chronic condition (cardiac, renal, etc.) that requires long-term anti-hypertensive therapy. Start weaning Labetalol first if on multiple anti-hypertensive medications. Do not decrease dose more frequently than every 2 days

Nifedipine XL

(decrease doses every

2-3 days as tolerated)

Labetalol

(decrease doses every 2-3 days as tolerated)

Labetalol TID

Nifedipine 60mg BID (max dose)

Labetalol BID

(Same Dose)

Nifedipine 90mg daily

Labetalol (decrease by 200mg increments (Ex: 800mg to 600mg)

Nifedipine 60mg daily

Labetalol 200mg BID

Nifedipine 30mg daily

Stop medication

Stop medication

Goal BP <120/<80 mmHg
